# Uptake of genetic counseling and testing in a clinic‐based population of women with breast cancer

**DOI:** 10.1002/cam4.4684

**Published:** 2022-03-23

**Authors:** Alexandra Wehbe, Mark Manning, Hadeel Assad, Kristen S. Purrington, Michael S. Simon

**Affiliations:** ^1^ Wayne State University School of Medicine Detroit Michigan USA; ^2^ Population Studies and Disparities Research Program Barbara Ann Karmanos Cancer Institute Detroit Michigan USA; ^3^ Department of Psychology Oakland University Rochester Hills Michigan USA; ^4^ Department of Oncology Barbara Ann Karmanos Cancer Institute Detroit Michigan USA

**Keywords:** breast cancer, genetic counseling, genetic testing, health care disparities, hereditary cancer syndromes

## Abstract

**Background:**

The study was conducted to evaluate racial differences in referral and uptake of genetic counseling (GC) in a clinic‐based population of women with breast cancer.

**Methods:**

Medical records of 150 breast cancer patients at the Karmanos Cancer Institute were reviewed to determine eligibility for GC according to National Comprehensive Cancer Network guidelines, GC referral rates, and appointment completion rates. Logistic regression was used to assess the relationship between demographic and clinical factors and GC eligibility and referral.

**Results:**

The mean age at diagnosis was 57.1 (SD 12.6) and 66% of the women were Black. There were 91 women (60.7%) eligible for GC and of those, 54 (61.4%) were referred. After multivariable analyses, factors associated with reduced eligibility were older age at diagnosis (OR = 0.91, 95% CI [0.87,0.95]) and Black race (OR = 0.37, 95% CI [0.15, 0.96]). After additional multivariable analysis, eligibility was associated with an increased likelihood of referral (OR = 5.97, 95% CI [2.29, 15.56]), however, Medicare versus private insurance was associated with a lower likelihood for referral (OR = 0.32, 95% CI [0.12–0.80]. Of those referred, 49 (76.6%) completed an appointment, and 47 had genetic testing. Women with Medicare were also less likely to complete an appointment. Race had no impact on referral or appointment completion.

**Conclusions:**

There were no racial differences in GC referral or appointment completion in a clinic‐based sample of women with breast cancer. Further interventions are needed to promote increased referral and appointment completion for women with breast cancer who are eligible for GC.

## Graphical abstract

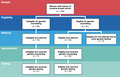

## 1 INTRODUCTION

Breast cancer is the most common cause of cancer and the second leading cause of cancer‐related death among women in the USA.^1^ There are approximately 3.8 million women in the USA with a history of breast cancer, 10% of which may be attributed to heritable mutations, most commonly *BRCA1 and BRCA2*.^2^ Nearly 940,000 women in the USA have hereditary breast and ovarian cancer syndrome (HBOC) by virtue of carrying a pathogenic variant in the *BRCA1* or *BRCA2* genes, however, it is estimated that only 5% of these women are aware of their carrier status.^3^ Furthermore, less than one in five eligible individuals with a personal history of breast cancer have undergone cancer genetic testing.^4^ Underutilization of genetic testing negatively impacts detection and preventative care services, which can substantially reduce future cancer risk and cancer‐associated mortality.^5^ Given known racial and ethnic disparities in breast cancer risk and mortality,^6^ it is important to examine whether there are similar disparities in genetic counseling (GC) referrals and uptake by racial and ethnic group.The National Comprehensive Cancer Network (NCCN) has established guidelines by which individuals may qualify for genetic testing based on their personal and/or family history of cancer.^7^ However, according to a recent population‐based study, over 70% of eligible women with breast cancer have never discussed genetic testing with a health care provider.^8^, ^9^ Recent studies suggest there are racial and ethnic disparities in awareness and utilization of genetic testing with several studies demonstrating substantially lower rates of referral for GC and testing among Black women compared to White women**.** ^10^, ^11^, ^12^, ^13^, ^14^ The disparity in referrals is even more troubling considering studies suggest rates of GC attendance following referrals are similar between Black and White patients.^13^ In other words, Black patients are just as likely to attend GC when referred, further highlighting the significant role of health care providers' referrals in promoting guideline concordant GC and genetic testing.

To examine overall rates and between‐race differences in eligibility for GC, referrals, clinic attendance, and genetic testing, we performed a medical record review of women with breast cancer who are followed at an urban comprehensive cancer center. Referral patterns and utilization of cancer GC and testing services were evaluated among women who met NCCN criteria for genetic testing. Also, demographic and clinical factors associated with eligibility for genetic testing, GC referral, and appointment completion were assessed. We specifically probed for between‐race (i.e., Black vs. White) differences given concerns gleaned from the literature and given that these are the modal racial groups seen in our comprehensive cancer center.

## 2 METHODS

### 2.1 Study population

The study population consisted of 150 women with invasive breast cancer who were seen in 2018 at the Karmanos Cancer Institute (KCI), one of the 51 National Cancer Institute recognized comprehensive cancer centers. Fifty charts were sequentially selected for medical record review from clinic schedules of each of three breast cancer medical oncologists starting chronologically with patients scheduled after January 1, 2018. Data were collected from the KCI medical record system (Cerner), the patient administered health history questionnaire (HHQ), physician medical notes, as well as pathology and GC reports.

### 2.2 Measures

We extracted information from medical records for demographic and clinical factors hypothesized to predict GC eligibility, GC referral, and completion of genetic testing. Demographic factors included age at the time of breast cancer diagnosis, race, ethnicity, marital status, insurance type, access to reliable transportation, highest education received, support from family and friends, and whether the primary language was English. Except for age at diagnosis, demographic information was extracted from patients' self‐administered HHQs and electronic data available in the medical record.

Clinical factors included age at breast cancer diagnosis, family cancer history, breast cancer stage at the time of the medical record review, presence and location of metastases if applicable, and breast cancer prognostic markers (Estrogen Receptor (ER), Progesterone Receptor (PR), and Her2neu), which were extracted from the pathology reports and medical oncology notes.

For family cancer history, information was largely derived from the HHQs and GC notes (when applicable). In cases where the HHQ was not available, we referenced the medical oncology notes for additional family cancer history information. However, family cancer history was not available in any of these locations and thus unknown for 37 women, leaving 113 (75.3%) women with a known family cancer history in the entire sample for analysis.

### 2.3 Outcomes

Outcomes included eligibility for cancer GC, rate of referral for GC, and completion of GC appointments. Eligibility was determined based on the 2019 NCCN guidelines.^7^ Women were deemed eligible based on their own personal cancer history if they met any of the following criteria: Breast cancer diagnosis prior to age 50, regardless of family cancer history; triple‐negative breast cancer at or before age 60; two separate primary breast cancers. Family cancer history criteria used to determine eligibility included: diagnosis of breast cancer at or before age 50 with one or more blood relatives diagnosed with breast cancer at any age; family history of cancers linked to HBOC (i.e., ovarian cancer, male breast cancer, pancreatic cancer, high‐grade prostate cancer [Gleason score ≥7], metastatic prostate cancer, or melanoma); two or more close blood relatives with breast cancer at any age. Since we did not have uniform information on ages at diagnosis for family members, we assumed patients over age 50 at diagnosis with only one family member with breast cancer at unknown age did not meet eligibility criteria for GC.

To determine GC referral, we used information available in the clinic database on whether a cancer genetics appointment was scheduled as a proxy indicator for whether the patient was referred. Secondary outcomes included whether the genetics appointment were completed and completion of genetic testing. We also reported on results of genetic testing when applicable.

### 2.4 Statistical analysis

We examined the bivariate relationship between demographic and clinical characteristics (predictor variables) and eligibility for GC referral. Six women received genetic testing at another location, however only three of these women were included in our analysis because they were referred for GC at our center. We then examined the relationship between predictor variables and referral to GC stratified by eligibility status using chi‐square tests of association for categorical predictors. We also looked at the relationship between predictor variables and completion of a GC appointment. Given women who identified as Black or White constituted >95% of the sample, we restricted analyses of bivariate associations with race to these two racial groups. We used ANOVA to examine associations between predictor variables and referrals and completion of a genetics appointment for continuous variables (i.e., age at the time of breast cancer diagnosis). We assessed stages as four categories (stages I, II, III, and IV) when examining associations with eligibility for genetic risk evaluation; for other evaluations which assessed referral and completion of appointments, we stratified stage as early stage (stages I‐III) versus late stage (stage IV). We refrained from statistical tests for relationships involving appointment completion given the small sample size.

We used multivariate logistic regression to assess unique predictors of eligibility and referrals to genetic testing. For the logistic regression models, all exclusion criteria used for the univariate analyses were applied to the multivariate model; hence, analyses were restricted to Black or White women who were neither of Arabic nor Hispanic ethnicity, for whom we had breast cancer stage and insurance data.

## 3 RESULTS

### 3.1 Description of the study population

Table 1 presents the distribution of sociodemographic and clinical factors of the study population stratified by eligibility for genetic risk evaluation as determined by NCCN criteria. The mean age at diagnosis was 57.1 years (*SD* = 12.6) and most of the cohort was Black (66.0%) followed by White (29.3%), and other racial or ethnic groups (4.7%). In addition, 35.3% of women were single, with 31.3% were married, 16.7% divorced or separated, and 16.7% widowed. Regarding education, 49% reported a college degree, 32.0% a high school degree only, 6.0% a grade school degree, 1.0% a doctoral degree or equivalent, and 12.0% listed their highest education as “other.” On the HHQ, most women (96.0%) reported support of family and friends during their cancer care, 98.0% reported English as their primary language, and 84% had reliable transportation for their oncology appointments (not in table). Most women had early‐stage disease (68.7%), and about half had Medicare (44.7%) or Medicaid (8.0%) at the time of medical record review. Of the 113 women with a known family cancer history, 43.4% had family cancer history patterns of HBOC‐related cancers making them eligible for GC per NCCN guidelines. Among the 148 women with tumor phenotype data available, 70.7% were ER+ and or PR+ but HER2Neu negative, 19.3% were HER2Nue positive, and 8.7% were triple negative.

**TABLE 1:** Demographic and clinical characteristics of study population stratified by eligibility status for genetic counseling

Characteristic	Total	Eligibility status	
		Ineligible	Eligible
Total (percent)		59 (39.3%)	91 (60.7%)
Mean Age at diagnosis (years)^a^	57.1	64	52.6
Race (percent)^b^			
Black	99 (66.0%)	45 (45.5%)	54 (54.5%)
White	44 (29.3%)	10 (25.0%)	30 (75.0%)
Asian	4 (2.7%)	—	—
American Indian	2 (1.3%)	—	—
Unknown	1 (0.7%)	—	—
Insurance type (percent)			
Private	70 (46.7%)	19 (27.1%)	51 (72.9%)
Medicare	67 (44.7%)	37 (55.2%)	30 (44.8%)
Medicaid	12 (8.0%)	3 (25.0%)	9 (75.0%)
No insurance	1 (0.7%)	1 (100.0%)	0 (0.0%)
Cancer stage (percent N)^c^			
I	52 (34.7%)	22 (42.3%)	30 (57.7%)
II	35 (23.3%)	14 (40.0%)	21 (60.0%)
III	16 (10.7%)	5 (31.3%)	11 (68.8%)
IV	47 (31.3%)	18 (38.3%)	29 (61.7%)

^a^ *F*_1,146_ = 35.79, *p* < 0.001). χ^2^(2) = 12.45, *p* = 0.002.

^b^ Note that subsequent analyses that examine racial differences will be restricted to Black and White women due to small subsample size.

^c^ There was no significant relationship between cancer stage and eligibility.

### 3.2 Univariable analyses of eligibility and referral for genetic counseling

Out of 150 women, 91 (60.7%) women were eligible for GC based on NCCN criteria. As shown in Table 1, factors associated with eligibility included younger age at diagnosis (52.6 vs. 64, *F*_1,146_ = 35.79, *p* < 0.001), self‐identification as White versus Black (75.0% vs. 54.5%, χ^2^[1] = 4.99, *p* = 0.026) and having Medicaid or Private insurance versus Medicare (75.0% and 72.9%, vs. 44.8%, respectively, χ^2^ [2] = 12.45, *p* = 0.002). There was no significant relationship between cancer stage at diagnosis and eligibility.

Table 2 shows factors associated with referral to GC stratified by NCCN eligibility status. Of the women eligible for GC, there were 54 who were referred (61.4%) and of those who were ineligible, there were 10 (16.9%) that were referred. Younger women were more likely to be referred (55.6 vs. 60.2; *F*_1,143_ = 3.96, *p* = 0.049), even after adjusting for the fact that younger women were more likely to be eligible (53.4 vs. 62.4; *F*_1,143_ = 14.98, *p* < 0.001). Among eligible women, those with Medicare were least likely to be referred (40.0%) compared to Medicaid (71.4%) or private insurance (72.0%), *p* < 0.015. There were no significant associations between insurance type and referral for ineligible women. Based on cancer stage at the time of the medical record review, more women with early‐stage disease were referred compared to stage IV (67.8% vs. 48.3%, *p* = 0.077).

**TABLE 2:** Factors associated with referrals to genetic counseling stratified by NCCN eligibility status

	Ineligible		Eligible	
	Genetic appointment referral		Genetic appointment referral	
	No (Percent N)	Yes (Percent N)	No (Percent N)	Yes (Percent N)
Total^a^	49 (83.1%)	10 (16.9%)	34 (38.6%)	54 (61.4%)
Mean age (years)	64.8	59.9	55.5	51.2
Race				
Black	37 (82.2%)	8 (17.8%)	22 (41.5%)	31 (58.5%)
White	9 (90.0%)	1 (10.0%)	12 (42.9%)	16 (57.1%)
Insurance type^b^				
Medicaid	2 (66.7%)	1 (33.3%)	2 (28.6%)	5 (71.4%)
Medicare	33 (89.2%)	4 (10.8%)	18 (60.0%)	12 (40.0%)
Private	14 (73.7%)	5 (26.3%)	14 (28.0%)	36 (72.0%)
Cancer stage				
Early (stages I‐III)	35 (85.4%)	6 (14.6%)	19 (32.2%)	40 (67.8%)
Late (stage IV)	14 (77.8%)	4 (22.2%)	15 (51.7%)	14 (48.3%)

^a^ χ^2^(1) = 28.4, *p* < 0.001.

^b^ Note: In table above, statistically significant difference in insurance type only among those who were eligible (χ^2^(2) 8.42, *p* = 0.015).

### 3.3 Multivariable analyses of eligibility and referral to genetic counseling

Table 3 shows results of logistic regression models of eligibility and referral to GC. As shown in univariable analyses, women who were older at time of diagnosis were less likely to be eligible (age OR = 0.91, 95% CI [0.87,0.95]) as were Black women compared to White women (OR = 0.37, 95% CI [0.15, 0.95]), however after multivariable adjustment insurance status was no longer a predictor of eligibility (OR = 1.07 for Medicaid vs. Private, 95% CI [0.18, 6.47]) (OR = 0.98 for Medicare vs. private, 95% CI [0.41, 2.37]).

**TABLE 3:** Logistic regression results for models predicting eligibility and referral for genetic counseling

Outcomes	Eligibility (*N* = 139)			Referred (*N* = 136)		
	OR	Lower 95% CI	Upper 95% CI	OR	Lower 95% CI	Upper 95% CI
Age at Diagnosis^a^	0.91^**^	0.87	0.95	0.98	0.94	1.02
Black^b^	0.37^*^	0.15	0.96	1.35	0.55	3.33
Breast Cancer Stage 4^c^	0.54	0.22	1.32	0.44^†^	0.17	1.14
Insurance Type^d1^						
Medicaid	1.07	0.18	6.47	1.36	0.28	6.73
Medicare	0.98	0.41	2.37	0.32^*^	0.12	0.80
Eligibility	—	—	—	5.97^**^	2.29	15.56

Note: ** = *p* < 0.01, * = *p* < 0.05, † = *p* < 0.10.

^a^ Mean centered age.

^b^ Reference = White.

^c^ Reference = Breast Cancer stage I–III.

^d^ Reference = Private insurance; 1: Omnibus categorical Χ^2^ = 0.01 (2), *ns* and 6.53 (2), *p* < 0.05 for Eligibility and Referred outcomes, respectively.

After multivariable analysis, prior eligibility was a significant predictor of GC referral (OR = 5.97, 95% CI [2.29, 15.56]). Additionally, compared to private insurance, even after adjustment for age, women with Medicare were less likely to be referred (OR = 0.32, 95% CI [0.12, 0.80]). Neither age at medical record review, race, stage, nor Medicaid insurance were significant predictors for referral.

### 3.4 Completion of genetic counseling appointment

Table 4 describes factors associated with completion of a GC clinic appointment among women referred for GC. Of 64 women referred for a genetics appointment, 49 (76.6%) completed their appointment, of which 42 were eligible for GC and seven were ineligible. Similar percentages of Black and White women completed their appointments (74% and 77%, respectively). Despite stage IV disease being negatively associated with referral, 83% of women with stage IV disease completed their appointment compared with 74% of women with stages I‐III. Fifty‐six percent of women with Medicare completed their GC appointment compared to 83% with Medicaid and 83% with private insurance. There was no apparent difference in age at diagnosis for women who completed or did not complete their appointment.

**TABLE 4:** Factors associated with completion of a genetic counseling appointment among women referred for genetic counseling

	Attended genetic counseling appointment	
	No (Percent N)	Yes (Percent N)
Genetic counseling eligibility		
Ineligible	3 (30%)	7 (70%)
Eligible	12 (22%)	42 (78%)
Race		
Black	10 (26%)	29 (74%)
White	4 (24%)	13 (77%)
Cancer stage		
Early‐stage (stages I‐III)	12 (26%)	34 (74%)
Late‐stage (stage IV)	3 (17%)	15 (83%)
Insurance type		
Medicaid	1 (17%)	5 (83%)
Medicare	7 (44%)	9 (56%)
Private	7 (17%)	34 (83%)
Mean age at diagnosis (years)	52.7	52.5

Overall, 47 women had genetic testing, including five who did not meet NCCN eligibility criteria. This represents 95.9% of women who completed an appointment, 46.2% of eligible women and 31.3% of the entire study sample. The genetic test results showed that of those tested, 28 women (59.6%) were negative for a pathogenic variant, four (8.5%) had a variant of unknown significance (VUS), and four (8.5%) were positive (results were unavailable for one woman). Of the five ineligible women who had genetic testing, one had a VUS and four tested negative for a pathogenic variant. Women with pathogenic variants were found to have variants in *BRCA1, BRCA2, MUTYH, and NTHL1*. VUS was identified in *BRCA1, BRCA2, MLH1, MRE11A, MUTYH, NBN, PALB2, RAD51C, SDHA, MSH6, and RAD50* genes. The individual with the pathogenic *MUTYH* variant was a heterozygous carrier and had no known family history of colonic polyposis. Further, the individual with the pathogenic *NTHL1* variant had no known family history of colonic polyposis.

## 4 DISCUSSION

In a sample of 150 women with breast cancer treated at a large, urban comprehensive breast center, almost two‐thirds (60.7%) of women were eligible for GC based on 2019 NCCN guidelines; however, of the eligible women, only 61.4% were referred for a genetics appointment. Once referred, only 77.8% of eligible women completed their appointments, which represents only 46.2% of the original eligible cohort. Of those who had subsequent genetic testing (96% of those who kept their appointments) the rate of a positive pathogenic variant was 8.4%. While Black women were less likely than White women to be eligible for GC, there were no racial differences in the referral to GC or completion of appointments once referred. We found that Medicare insurance was inversely associated with GC referral even after accounting for age and eligibility, and descriptive evidence suggested a negative association with appointment completion.

Our findings revealing just over half (61.4%) of the women eligible for GC were ultimately referred is consistent with other studies showing the underutilization of GC referrals among women at high risk for HBOCs.^15^, ^16^ These findings underscore the critical need to identify system‐level and physician‐level barriers to refer eligible cancer patients for GC. Forty‐seven women (31.3%) of our entire sample underwent genetic testing, including five who were ineligible, indicating an even lower proportion of correctly identified women who ultimately underwent genetic testing. Failure to refer eligible cancer patients ultimately denies them of information essential to estimating their own future risk of recurrence and secondary diagnoses.^5^ Failure to refer eligible patients also results in missed opportunities for cascade screening of blood relatives, denying relatives the chance to learn about and manage their potential inherited risks. In that cascade screening can be a cost‐effective public health strategy,^17^, ^18^ and that the potential yield of pathogenic variants among cancer patients is likely higher than the general population, increasing referrals of eligible cancer patients for GC may have significant benefits at the level of the patient, family, and public health.

In our analysis, type of medical insurance affected referral and appointment completion rates. Even after adjustment for age, women with Medicare were less likely to be referred and less likely to complete their appointment compared to women with Medicaid or private insurance. This could be related to financial toxicity or higher comorbidities seen in women with Medicare, however these factors were not evaluated in our study.

Despite the presence of treatment implication for metastatic triple‐negative breast cancer associated with *BRCA1/2 mutations,* we found that women with stage IV disease were somewhat less likely to be referred for their appointments than women with earlier stage disease, although this was only significant in univariate analysis. Once a referral was made, even more women with stage IV disease completed their appointment compared to women with earlier stage disease.

Consistent with prior studies, there were no racial differences in the proportion of eligible women who were referred for an appointment, or for those who completed a GC appointment.^13^, ^14^, ^19^, ^20^, ^21^, ^22^, ^23^ Once referred, Black women were just as likely as White women to utilize GC services. Other literature which suggests lower GC attendance rates for Black compared to White patients suggest it is important to intervene in a more proximal part of the referral process; that is, to evaluate barriers to GC referral, such as lack of physician recommendation.^10^, ^20^, ^22^ Upstream interventions could include physician education or system‐level interventions such as trained nurse navigators who may effectively identify patients eligible for GC.^24^ Our findings highlight that when there is no upstream disparity, downstream disparities are less likely. This underscores the importance of GC referrals, and the call for adherence to genetic risk‐evaluation guidelines in the referral process.

A limitation of our analysis was the small sample size and that our data came from a single institution and included patients seen by only three medical oncologists. Furthermore, the data derived for this analysis were obtained through medical record review and was restricted to information provided by patients who filled out an HHQ or what was documented in the medical record. It is possible more women were eligible for genetic testing than were detected, but were presumed ineligible if their family history was unknown. Furthermore, missing information on age at diagnosis of affected family members could have resulted in underrepresentation of eligibility for GC and testing. Additionally, documentation of a patient appointment in the medical record system at the KCI was used as a proxy for a referral to GC, however, physicians could have referred patients for GC at other sites that were not captured in our review. Strengths of our study are that they represent the type of eligibility information and conditions typically seen in an outpatient setting, and provide insight as to patterns of eligibility, referrals, and appointment completion seen in a hospital‐based oncology practice.

These analyses serve as a needs assessment for a future‐planned randomized trial of patient and physician‐based interventions designed to improve uptake of cancer GC and testing. Our results showing that only 61.4% of eligible women were referred for GC, suggest a gap between the need for genetic testing and utilization of it. This is seen through the incremental decrease in the percentage of eligible women receiving testing at the various steps between breast cancer diagnosis, GC appointment, and having a genetic test completed. Every eligible patient that does not undergo genetic testing is a missed opportunity for tailored cancer treatment, surveillance, and screening for patients and their family members. These results are consistent with findings from the few studies that have documented a paucity of GC among eligible cancer patients, and further supports the conclusion that efforts must be made at each step of the referral process to ensure patients are receiving these services.

## CONFLICT OF INTEREST

The authors have no conflict of interest to disclose.

## AUTHOR CONTRIBUTIONS

Alexandra Wehbe: Conceptualization, investigation, methodology, data curation, project administration, writing – original draft, writing – review, and editing. Mark Manning: Formal analysis, investigation, writing – review, and editing. Hadeel Assad: Methodology, reviewing, and editing. Kristen Purrington: Methodology, reviewing, and editing. Michael S. Simon: Conceptualization, investigation, methodology, supervision, writing – review, and editing.

## ETHICAL APPROVAL AND INFORMED CONSENT STATEMENT

This study was approved by the Institutional Review Board (IRB). According to IRB guidelines, neither approval from the ethics committee nor informed consent from the study populations is required for this study.

## Data Availability

The data that support the findings of this study are available from the corresponding author upon reasonable request.
